# Hyponatremia due to adrenal insufficiency after a *Mamushi* bite: a case report

**DOI:** 10.1186/s12902-023-01466-4

**Published:** 2023-10-11

**Authors:** Ryu Sugimoto, Tsuneaki Kenzaka, Hogara Nishisaki

**Affiliations:** 1 Department of Internal Medicine, Hyogo Prefectural Tamba Medical Center, 2002-7 Iso, Hikami-Cho, Tamba, 669-3495 Japan; 2 Division of Community Medicine and Career Development, Kobe University Graduate School of Medicine, Kobe, Hyogo Japan

**Keywords:** Hyponatremia, Sodium, Snake bites, Adrenal insufficiency, Empty sella, Snake venom

## Abstract

**Background:**

*Mamushi* bites are the most common venomous snake bites in Japan, with known complications including rhabdomyolysis and acute kidney injury; however, adrenal insufficiency as a result of snake bites has not been previously reported. We report a case of empty sella with transient adrenal insufficiency during hospitalization for a *Mamushi* bite.

**Case presentation:**

An 84-year-old man was admitted to our hospital with a *Mamushi* bite on the right fifth finger. Serum sodium (Na) level remained in the normal range. On the ninth day of admission, he developed hyponatremia, with a serum Na level of 114 mEq/L and serum cortisol level of 4.0 μg/dL (reference value 4.5–21.1 μg/dL). His serum Na level was restored within the normal range after administration of corticosteroids with 3% NaCl solution. Both rapid adrenocorticotrophin and corticotropin-releasing hormone loading tests showed low cortisol response. Based on the results of the hormone loading tests, a diagnosis of pituitary adrenal insufficiency was made. Contrast-enhanced pituitary magnetic resonance imaging (MRI) showed primary empty sella. After discontinuation of corticosteroids, the hyponatremia did not recur, and the patient was discharged on the 24th day of hospitalization. After discharge, the patient visited an outpatient clinic, but hyponatremia recurrence was not observed.

**Conclusions:**

This is the first report of hyponatremia due to pituitary adrenal insufficiency during hospitalization for a *Mamushi* bite in a patient with empty sella. When hyponatremia occurs during hospitalization for a *Mamushi* bite, cortisol measurement, hormone loading test, and head MRI should be performed to search for pituitary lesions because of the possibility of adrenal insufficiency caused by snake venom.

## Background

*Mamushi* (*Gloydius blomhoffii*), the most common venomous snake in Japan, is found throughout Japan, except in Okinawa. Approximately 1000 cases of *Mamushi* bites are reported annually, with an estimated mortality rate of approximately 1% [1]. Known complications of *Mamushi* bites include acute kidney injury, hypovolemic shock, and rhabdomyolysis [2]. Although rare, fatal complications such as respiratory failure, gastrointestinal hemorrhage, and intestinal necrosis have also been reported [3].

To date, there have been no case reports of adrenal insufficiency following a *Mamushi* bite. Empty sella is defined as an enlarged subarachnoid space within the sella turcica [4] and is associated with various degrees of hormone deficiency [5]. In this report, we present a case of empty sella with transient adrenal insufficiency during hospitalization for a *Mamushi* bite.

## Case presentation

### Case

An 84-year-old Japanese man was bitten by a *Mamushi* on the right fifth finger and presented to our emergency department. He had a history of benign prostatic hyperplasia and an overactive bladder. He was taking mirabegron at a dosage of 50 mg/day and had an oxybutynin patch that delivered a dose of 73.5 mg/day. The patient was treated on surgical admission. On the second day of hospitalization, the bite reached Grade 4 (redness and swelling of the whole extremity) [6]. Creatine kinase (CK) level was maximal at 1770 U/L on the fourth day of admission. Cephalantin (10 mg/day), acetate ringer (1000 mL/day), and maintenance fluid (sodium 35 mEq/L, potassium 20 mEq/L, and glucose 4.3% isotonic solution [1000 mL/day]) had been administered since the second day of admission. The total in–out balance had roughly passed zero.

On the eighth day of hospitalization, vomiting occurred. In the early hours of the ninth day of hospitalization, the patient experienced restlessness, and blood tests revealed hyponatremia. The patient was transferred to the department of internal medicine.

### Investigations

At the time of transfer to the department of internal medicine, the patient’s vital signs were as follows: consciousness level of 13 points (E3V4M6) on the Glasgow Coma Scale, blood pressure of 142/79 mmHg, pulse rate of 59 beats/min, respiratory rate of 24 breaths/min, SpO_2_ of 97% (room air), and body temperature of 37.2 °C in the axilla. Physical examination revealed no rigidity of the neck, no enlarged cervical lymph nodes, and clear respiratory sounds and no heart murmur on chest auscultation. The abdomen was flat and soft with no tenderness. No edema of the lower legs was observed, and tenderness, swelling, and heat were present from the right upper arm to the dorsum of the right hand. The findings in the right upper extremity remained unchanged from those on the previous day.

Table 1 shows the laboratory findings at the time of transfer to the department of internal medicine. Blood tests showed that the CK level was elevated again to 2267 U/L. The serum Na level was 114 mE/L, and serum osmolality was 237 mOsm/L. Urinalysis revealed a urine specific gravity of 1.020, urinary Na level of 195 mEq/L, and urine osmolality of 613 mOsm/L. Despite the hyponatremia, the urine was hypertonic.

**Table 1:** Laboratory data on the ninth day of admission

Parameter	Recorded value	Standard value
White blood cell count	7,900/µL	4500–7500/µL
Neutrophils	77.9%	42–74%
Lymphocytes	10.3%	18–50%
Eosinophils	0.5%	0–7%
Hemoglobin	12.2 g/dL	11.3–15.2 g/dL
Platelet count	12.8 × 10^4^/µL	13–35 × 10^4^/µL
Prothrombin time / International normalized ratio	0.97	0.80–1.20
Activated partial thromboplastin time	36.0 s	26.9–38.1 s
D-dimer	5.6 μg/mL	< 1.0 μg/mL
C-reactive protein	0.71 mg/L	≤ 0.14 mg/dL
Total protein	6.0 g/dL	6.9–8.4 g/dL
Albumin	3.5 g/dL	3.9–5.1 g/dL
Total bilirubin	2.3 mg/dL	0.2–1.2 mg/dL
Aspartate aminotransferase	80 U/L	11–30 U/L
Alanine aminotransferase	39 U/L	4–30 U/L
Lactase dehydrogenase	506 U/L	109–216 U/L
Creatine kinase	2,267 U/L	40–150 U/L
Blood urea nitrogen	14.8 mg/dL	8–20 mg/dL
Creatinine	0.52 mg/dL	0.63–1.03 mg/dL
Sodium	114 mEq/L	136–148 mEq/L
Potassium	4.5 mEq/L	3.6–5.0 mEq/L
Chloride	85 mEq/L	101–108 mEq/L
Glucose	88 mg/dL	70–109 mg/dL
Plasma osmolarity	237 mOsm/L	275–290 mOsm/L
Plasma adrenocorticotrophin hormone	34.9 pg/mL	7.2–63.3 pg/mL
Serum cortisol	4.0 µg/dL	4.5–21.1 µg/dL
Urinalysis		
Specific gravity	1.020	
Protein	±	
Blood	1 +	
Urinary sodium	195 mEq/L	
Urinary potassium	35 mEq/L	
Urinary chloride	159 mEq/L	
Urinary creatinine	44.5 mg/dL	
Urinary osmolarity	613 mOsm/L	

### Differential diagnosis

**Fig. 1:**
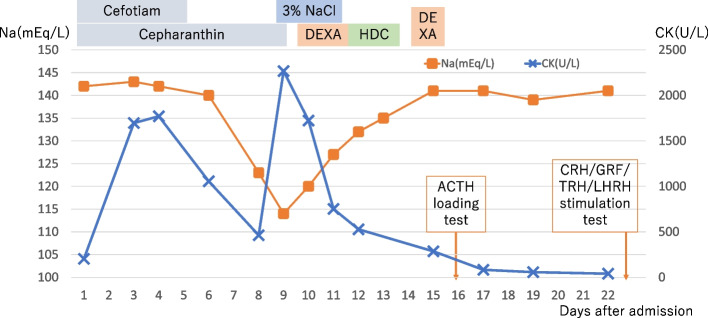
Chart showing the patient’s clinical course. DEXA: dexamethasone 6.6 mg/day, HDC: hydrocortisone 100 mg/day, NaCl: sodium chloride, CK, creatine kinase, ACTH: adrenocorticotropic hormone, CRH: corticotropin-releasing hormone, GRF: growth hormone-releasing factor, TRH: thyrotropin-releasing hormone, LHRH: luteinizing hormone-releasing hormone

Figure 1 shows the clinical course of the patient after admission. We determined that the CK re-elevation was due to hyponatremia. Severe symptomatic hyponatremia was corrected with administration of 3% sodium chloride solution. On the 10th day of admission, the serum Na level improved to 120 mEq/L, and the level of consciousness improved as the serum Na level improved. Since the blood cortisol level was low at 4.0 µg/dL (reference value 4.5–21.11 µg/dL), adrenal insufficiency was suspected, and administration of corticosteroids (dexamethasone 6.6 mg/day, then hydrocortisone 100 mg/day) was initiated on the 10th day of admission. On the 11th day of admission, the 3% sodium chloride solution was discontinued, and on the 15th day of admission, the corticosteroids were discontinued; however, the serum Na level did not decrease. An adrenocorticotrophin hormone (ACTH) loading test was performed on the 16th day of admission. Pituitary contrast-enhanced magnetic resonance imaging (MRI) and a corticotropin-releasing hormone / growth hormone-releasing factor / thyrotropin-releasing hormone / luteinizing hormone-releasing hormone (CRH/GRF/TRH/LHRH) stimulation test was performed on the 19th and 23rd day of admission, respectively. The results of the ACTH loading test and CRH/GRF/TRH/LHRH stimulation test are shown in Table 2. The peak blood cortisol levels after the ACTH loading and CRH/GRF/TRH/LHRH stimulation tests were below 18 µg/dL, and the peak ACTH level after the CRH/GRF/TRH/LHRH stimulation test was less than two-folds the basal value; thus, a diagnosis of pituitary adrenal insufficiency was made [7].

**Table 2:** Results of the hormone loading tests

**ACTH loading test**								
**Parameter**		**Time (min)**						
	**Standard value**	**0**		**30**			**60**	
Serum cortisol (µg/dL)	4.5–21.1	0.3		10.1			14.5	
**CRH/GRF/TRH/LHRH stimulation test**								
**Parameter**		**Time (min)**						
	**Standard value**	**0**	**30**		**60**	**90**		**120**
Plasma ACTH (pg/mL)	7.2–63.3	77.5	71.0		98.5	104.0		98.5
Serum cortisol (µg/dL)	4.5–21.1	12.1	10.3		12.8	16.2		13.1
Serum TSH (µU/mL)	0.35–4.94	1.24	11.1		10.2	9.2		6.3
Serum GH (ng/mL)		0.73	13.8		11.5	7.0		3.8
Serum prolactin (ng/mL)	3.6–12.8	7.0	63.6		58.2	41.7		32.3
Serum LH (mU/mL)	0.8–5.7	4.5	18.3		22.8	22.0		23.0
Serum FSH (mU/mL)	2.0–8.3	12.1	15.1		17.1	16.2		19.3

*ACTH* adrenocorticotropic hormone, *CRH* corticotropin-releasing hormone, *GRF* growth hormone-releasing factor, *TRH* thyrotropin-releasing hormone, *LHRH* luteinizing hormone-releasing hormone, *LH* luteinizing hormone, *FSH* follicle-stimulating hormone

**Fig. 2:**
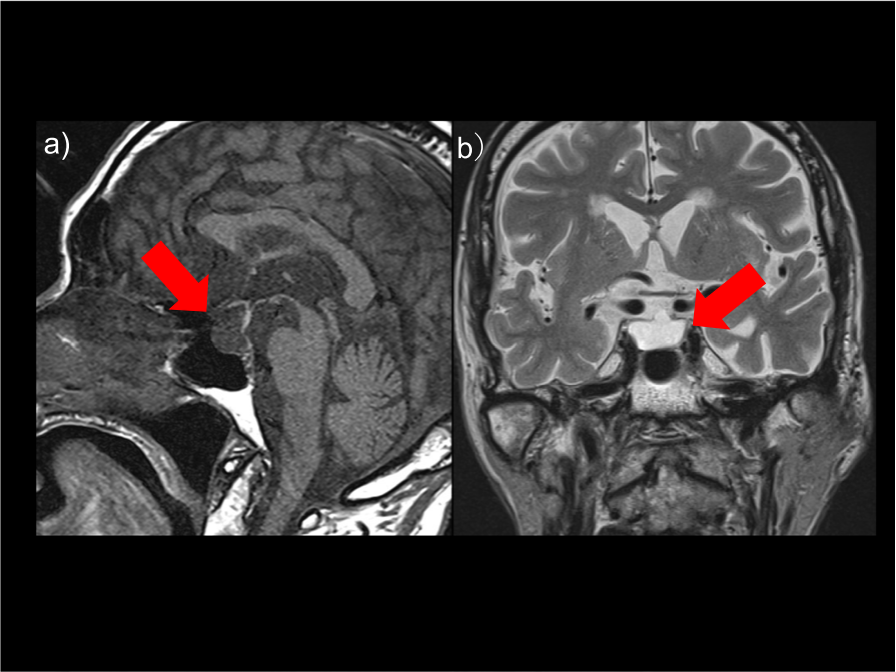
Contrast-enhanced pituitary magnetic resonance imaging. **a** T1-weighted image – sagittal section; (**b**) T2-weighted image – coronal section shows empty sella

Contrast-enhanced MRI of the pituitary gland is shown in Fig. 2. The sella turcica was markedly enlarged and filled with cerebrospinal fluid.

### Outcome and follow-up

After discontinuation of corticosteroids, the hyponatremia did not recur, and the swelling in the right upper extremity improved. The patient was discharged on the 24th day of admission. He visited the outpatient clinic 9 and 16 days after discharge, and there was no hyponatremia relapse. The patient has not experienced hyponatremia in the three years since the *Mamushi* bite.

## Discussion and conclusions

This is the first report of a case of hyponatremia due to pituitary adrenal insufficiency after a *Mamushi* bite, and close examination revealed empty sella. To the best of our knowledge, this is the first report of adrenal insufficiency after a *Mamushi* bite in the global literature.

In the present case, the onset of hyponatremia occurred notably later than did the peak swelling of the right upper limb and the peak CK level. The timing of hyponatremia manifestation does not align with the severity of the disease due to a *Mamushi* bite. The patient's cortisol response during the CRH/GRF/TRH/LHRH stimulation test conducted on the 23rd day of admission, when symptoms from the Mamushi bite had already improved, was low. Therefore, we concluded that the adrenal insufficiency in this case represents a distinct condition unrelated to the relative adrenal insufficiency seen in critically ill patients. Regarding the results of the ACTH loading test performed on the 16th day of admission, adrenal function could possibly be underestimated due to administration of dexamethasone on the previous day. However, given the significantly low initial cortisol levels and the fact that the CRH stimulation test was conducted more than one week after the last steroid administration, we believe that even if the ACTH loading test results were underestimated, it does not change the current diagnosis.

Hypopituitarism has been reported as a rare complication of Russell’s viper bites [8]. In a case series of acute hypopituitarism following a Russell’s viper bite [9], the median time from the bite to the onset of hypopituitarism was 9 days, which is similar to the onset time in our case. The mechanism of acute hypopituitarism after a Russell’s viper bite is described as follows: in the first stage, the pituitary gland becomes more vulnerable due to irritation from the direct action of the snake venom and swells due to capillary leak syndrome. In the second stage, microthrombosis due to disseminated intravascular coagulation (DIC), hypotension, and intracranial hypertension occurs, resulting in pituitary gland infarction and bleeding [10]. In general, the detailed components and physiological effects of snake venom are unknown, but because many components, such as phospholipase A2 and L-amino acid oxidase, are shared across species [2, 11], *Mamushi* bites can cause the same pathological conditions as Russell’s viper bites.

In this case, the patient’s condition was accompanied by primary empty sella, but there was no history of trauma, intracranial surgery, or radiotherapy, and the condition was judged to be primary empty sella [4]. We concluded that the empty sella was present prior to the *Mamushi* bite. The etiology of a primary empty sella is not clearly understood, and various hypotheses have been proposed [4]. The causes include a defect in the sellar diaphragm, increased intracranial pressure, and changes in the pituitary gland volume. Increased intracranial pressure is reportedly involved in the development of empty sella, and a mechanism in which the subarachnoid space fits into the sella turcica due to increased intracranial pressure against the background of a defective or hypoplastic sellar diaphragm has been postulated [4, 12]. In the present case, there were no signs of hypotension, hemorrhage, or DIC; however, the swelling of the pituitary gland and the increased intracranial pressure caused by the snake venom may have further increased the pressure on the pituitary gland, which was already pressurized within the sella turcica, leading to hypopituitarism. The pituitary gland is one of the most vascularized tissues in the human body [13], and as part of the mechanism of Sheehan’s syndrome, which also causes hypopituitarism, a mechanism in which the pituitary gland, enlarged by pregnancy, presses on the superior pituitary artery has been postulated [14]. Based on the above considerations, we hypothesize that the patient with empty sella was originally vulnerable to compression and ischemia.

In the present case, the patient developed hypopituitarism after a *Mamushi* bite, but only a transient decrease in pituitary adrenal insufficiency was observed. The majority of patients who develop hypopituitarism in the acute phase of a Russell’s viper bite are reported to have hypopituitarism in the chronic phase [15, 16]. Russell’s viper bites have been reported to be 2.6–23 times more fatal than *Mamushi* bites [17, 18]. Since a *Mamushi* bite is milder than a Russell’s viper bite, we consider that the hypopituitarism in our case was transient. The prevalence of primary empty sella is reported to be 2–20% [19], and it is a fairly common condition; therefore, explaining this rare condition entirely by the presence of primary empty sella may be difficult [19]. Intraspecific geographic variation [17] and changes in venom composition with individual snake maturity [20] have also been reported, and the composition of the *Mamushi* venom in our case may be associated with the pathogenesis of the disease.

Adrenal insufficiency during hospitalization for *Mamushi* bite is very rare, but pituitary adrenal insufficiency may develop by the same mechanism as that involved in Russell’s viper bite, especially in patients with a fragile pituitary gland. If hyponatremia develops during hospitalization for *Mamushi* bite, pituitary adrenal insufficiency should be considered, and appropriate correction, corticosteroid-related tests, and head MRI should be performed.

In conclusion, we report for the first time a case of hyponatremia due to pituitary adrenal insufficiency following a *Mamushi* bite in a patient with empty sella. When hyponatremia develops during the course of a *Mamushi* bite, considering the possibility of adrenal insufficiency due to snake venom, cortisol measurement and loading tests should be performed, and head MRI should be performed to search for pituitary lesions.

## Data Availability

All data generated or analyzed during this study are included in this published article.

## Abbreviations

ACTH: Adrenocorticotrophin hormone
CK: Creatine kinase
CRH/GRF/TRH/LHRH: Corticotropin-releasing hormone / growth hormone-releasing factor / thyrotropin-releasing hormone / luteinizing hormone-releasing hormone
DIC: Disseminated intravascular coagulation
MRI: Magnetic resonance imaging
Na: Sodium
